# Response to Comment on “Comparing Physician Assistant and Nurse Practitioner Practice in US Emergency Departments, 2010–2017”

**DOI:** 10.5811/westjem.2022.8.58423

**Published:** 2022-09-14

**Authors:** Michael Darracq, Fred Wu

**Affiliations:** University of California, San Francisco, Department of Emergency Medicine, Fresno, California

Dear Editor:

We wish to express appreciation to the authors for their kind words regarding our research article.^1^ We also appreciate the opportunity to respond to the methodological concerns raised.

The authors raise the concern that in the data analysis subsection there are no details regarding the statistical methodology that is used to compare group differences. We feel that this is an incorrect statement. Proportions with 95% confidence intervals are an accepted and often recommended method of comparison between groups. We disagree that hypothesis testing, as suggested by the authors, is necessary as hypothesis testing and confidence intervals rely on the same underlying methodology. Statistical significance can be determined by *P*-values or confidence intervals. These two approaches always agree.^2^

In 2016, the American Statistical Association released a position paper describing the over reliance on *P*-values in medical research and considerable misuses and misconceptions regarding *P*-values. They also described the use of alternative methods that emphasize estimation over testing, such as confidence, prediction, or credibility intervals.^3^ We also chose to limit the number of statistical tests performed because of issues related to multiple comparisons. As more attributes are compared, the greater the likelihood of the groups being different on the basis of random sampling error alone.^4^ The few *P*-values reported are the result of the two-sample z test for proportion, the only statistical test that we, as nonprofessional statisticians, are aware of for comparing proportions. This was requested by the journal editors to support our statements regarding statistically significant differences.

The authors also cite a reference that describes ways to interpret a graph with error bars as it relates to confidence intervals and overlap in determining a *P*-value or statistical significance.^5^ No inference was performed on the basis of overlap of confidence intervals. As this was not the methodology that was used in the present study, any description of inability to determine intergroup difference is not relevant.

Thank you for your comments and time on this important topic.
